# Abstract of Proceedings of the Buffalo Medical Association

**Published:** 1868-05

**Authors:** T. M. Johnson

**Affiliations:** Sec’y.


BUFFALO

### Medical and Surgical Journal.

VOL. VII. MAY, 1868. No. 10.

### Original Communications.

---

ART. I.—*Abstract of Proceedings of the Buffalo Medical Association.*

TUESDAY EVENING, April 7th, 1868.

The meeting was called to order by the President. Members present—Drs. Eastman, White, Lockwood, Miner, Ring, Greene, Dayton, Diehl, Little, C. F. A. Nichell, Henry Nichell, Mackay, Gay, Samo, Phelps, Rochester and Johnson.

The minutes of the last meeting were read and approved.

The annual report of the Treasurer was read, and on motion of Dr. Johnson the report was adopted and referred to the auditing board.

DR. WHITE moved that a vote of thanks be tendered to Dr. T. T. Lockwood for the efficient manner in which he has discharged the duties of the office of Treasurer. Carried.

The Secretary's report was read, and by vote accepted and ordered placed on file.

DR. MINER moved that the thanks of the Association be tendered to the Secretary for the efficient and acceptable manner in which he has performed the duties of his office. Carried.

Election of officers being next in the order of business the following officers were elected for the ensuing year:

For President, - Dr. J. R. Lothrop.
" Vice President, - " T. T. Lockwood.
" Secretary, - " T. M. Johnson.
" Treasurer, - - " C. F. A. Nichell.
" Librarian, - " James B. Samo.

Dr. Gay read the following paper:

*Placenta Previa.*

February 8th, 1860, visited along with Dr. Storck a German female, aged 30 years, residing near Delaware Place, been flowing three days. A midwife had been in attendance for two days; pains had ceased for some time previous to our visit; patient quite exsanguinous; pulse feeble; attacks of syncope frequent. On digital examination found os fully dilated and the placenta presentiug left laterally. No hemorrhage at present time. The child was easily turned, the feet brought down, and the woman delivered. Forceps were used in delivery of the head. A great deal of blood had evidently been lost during the early stage of labor, but during the process of delivery but little additional blood escaped. This patient died fifteen days afterwards from puerperal fever.

Mrs. A., aged 28 years, English, had three children, no difficulty at their birth. This woman was very plethoric and limbs somewhat edematous. She had taken the upright position for her confinement and insisted upon her right to maintain this position during her labor. On examination found the os partially dilated and the placenta presenting left laterally; pains vigorous, and at every pain profuse hemorrhage. The emergency seemed pressing, and not knowing what better to do, resorted to venesection, and to my surprise and gratification, discovered that the venesection had entirely arrested the uterine hemorrhage, and as the presentation was normal, labor progressed rapidly until delivery was naturally accomplished, there being no post-partem hemorrhage. This case dates back to the time when venesection was more in vogue than it is at the present time. Date not recorded in my note-book.

Mrs. B., German, aged 26 years, the mother of three children. Visited this patient along with Dr. Hauenstein, August 15, 1867. The doctor had diagnosed placenta previa, before having made an examination, for during the latter weeks of utero-gestation, there had been frequent recurrence of hemorrhage, and at our visit the hemorrhage was considerable, so much so as to measurably prostrate the patient; pains feeble. The tampon had been introduced at the doctor's first visit. Dilatation to the extent of a crown-piece, os rigid. The placenta was centrally implanted; presentation of child not ascertained. In consultation we decided to deliver at once. Patient under influence of chloroform, Dr. H. brought down one of the legs and delivered a still-born child; the placenta immediately followed; much blood was lost, but patient appeared cheerful and hopeful. Pressure with both hands upon the abdomen was constantly maintained, but in spite of persistent pressure, the uterus dilated and continued to dilate until it mounted up above the umbilicus, when the flooding became copious and fatal. The patient at once became conscious of approaching death; soon became comatose without any apparent intermediate syncope, and died in an hour and a half after delivery of post-partem hemorrhage.

We decided then and there, that if so unfortnunate as to be ever called to another case of central implantation, that we would adopt another plan of procedure—a plan not recognized as authoritative. We had not long to wait, for on February 23d, another case presented itself, very nearly in the same locality of the city, and on April 4th, still another case, both of central implantation.

The first, a multipara, German, aged 38 years. Hemorrhage first occurred on January 23d, at eight months utero-gestation. The air-ball was at once used, and the vagina effectually tamponed. At precisely four weeks to a day after the first attack of hemorrhage she was again attacked with flooding, and was now at the completion of her full period of utero-gestation. Labor pains soon commenced.

The patient was visited at 5 A. M. The air-ball again placed and retained in situ and the abdomen tightly bandaged. We resolved to wait in this case to the extreme point of waiting for efforts of nature to accomplish what she might be able to accomplish unaided, save appropriate means for the arrest of hemorrhage, and we did thus wait even beyond the verge of apparent safety, and did thus delay until the os was fully dilated. We had ascertained from external palpation that the position of the child was transverse. Pains had been considerable at noon, but now, at night at nine o'clock there were none. Hemorrhage had been controlled by the tampon; at 9½ P. M. the tampon was removed; the os found fully dilated; entrance of the hand within the uterine cavity easily made, the patient having been previously anæsthetised; one foot seized, brought down, and when the breach became engaged, hemorrhage which had just before been considerable, was now arrested, and the further delivery, as the pains had become active, was left to the unaided efforts of nature. This woman gave birth to a living child, and had, herself, a good recovery. I should add that pressure was constantly made with both hands upon the abdomen, and maintained for some considerable time after delivery.

The other case occurred on April 4th; was the mother of three children, the youngest being nine years of age; the age of the patient was 40 years. Hemorrhage first occurred either at 8 or 8½ months utero-gestation. At 9 P. M. April 3d, copious hemorrhage commenced; at 2 o'clock A. M. April 4th, labor pains set in. The air-ball was promptly used; the patient bandaged tightly and hemorrhage was controlled. Visited the patient along with Dr. Hauenstein at 1 P. M. At this time there was some hemorrhage in spite of the tampon; pains regular; pulse feeble; nearly nominal in frequency; tendency to syncope. Examination had been previously made and central implantation diagnosed; dilation was sufficient to easily admit one finger. Advised continuance of treatment. 5 P. M. patient much in same condition; counseled delay. 8½ P. M. no material change; general condition of patient same; labor progressing; removed tampon; found dilatation of os about one-half completed; introduced air-ball; applied bandage, and was called again in haste at 3½ A. M. April 5th. There had been some hemorrhage in spite of the tampon during the past hour. The position of the child was found on palpation to be transverse, as in the other case; pulse feeble and frequent. Patient under the influence of chloroform the child was turned and delivered, the os having been completely dilated and entrance of the hand within the uterine cavity easily made. Mother and child both saved and doing well. Constant pressure with both hands, as in the former case, was maintained during and for some considerable time after delivery.

At 11 A. M. April 5th, no untoward symptoms.

I am permitted to allude to another case occurring in the practice of Dr. Hauenstein, which was fatal, and the doctor assures me that very little blood was lost. This was a case of placenta previa lateralis, and I am assured by the doctor who was in attendance from beginning to end of labor, that he had seen as much blood lost in an ordinary case of labor as he saw in this case. The child was turned and delivered.

It is worthy of note that six cases of placenta previa have occurred in the practice of Dr. H. in the space of the last eight months. Another fact worthy of note is, that three out of five cases seen by myself occurred within a few yards of each other.

These cases, their termination and management are suggestive, and incitive to thought. They suggest in regard to their management a suspension of pre-conceived views. They call to mind the principle long since laid down and acted upon generally by authors and practitioners of the danger of intermeddling midwifery, and suggest a greater reliance upon the provisions of nature and less dependence upon art. They also suggest that delay is not so much to be deprecated, and that it is not so hazardous as precipitous haste would be, and that if a woman must die within one or two hours after forcible entrance has been effected within her womb, and the child speedily delivered, that it may be better to wait for complete dilatation even if she must necessarily die a few hours later. In the one form of procedure the shock is much greater than in the other.

Taking these imperfectly reported cases as a basis for remarks, I have proposed to myself the task of making some clinical observations upon the subject of placenta previa, and have now in process of completion a paper which I hope to present to the Society at a future meeting.

Dr. White said:—Mr. President, the gentleman who has just taken his seat has introduced to the consideration of the members of this Society a subject of the gravest importance. Unavoidable hemorrhage, or placenta previa, though of rare occurrence, is sufficiently frequent, and the danger so imminent that it is of the first importance that every practitioner should be familiar with the best course to be pursued in its management. It is, in my opinion, therefore, our imperative duty to brand such crude doctrines as those just advanced, as heterodox, and disclaim on the part of this Society all endorsement of the treatment pursued in the cases related, and of the "new" rule of practice which the doctor finally recommends as the result of his experience. Not doubting that the sole purpose of the member, Dr. Gay, in writing the paper to which we have just listened, was the advancement of medical science, I shall feel free to speak plainly in my comments upon the points discussed without fear of giving offense or being suspected of having the least personal feeling. The proposition, however, to leave all cases of unavoidable hemorrhage to nature; or in other words, the medical attendant sitting by, supinely folding his hands and permitting the poor woman to flow until death comes to her relief, is too startling to permit it to go out to the world from this Society unchallenged.

Let us first examine for one moment some of the cases related by Dr. Gay and the treatment pursued by him producing results which impelled him to this "new" method. The first case lost so much blood before art interfered for her relief that she apparently never rallied from her exhausted and exsanguine condition. The reasons for this extraordinary delay are not very clearly stated, and perhaps as the woman was in the hands of a midwife during the early part of her labor, the only criticism which it would be fair to make is, that turning was resorted to too late to save the patient.

Proceeding to the second case, we find that having the unfortunate result from *delay* in the first case, staring him in the face, the doctor resolved not to be behindhand in this instance, and as he informs us proceeded "forcibly" to turn. I am astonished, Mr. President, to hear the word "forcible" used at this late day to describe any of the manipulations practiced for the delivery of the parturient. The patient died, and as no *post-mortem* is reported, it is impossible to determine the condition of the organs after this "forcible" resort to version of the child in utero.

In the third case, not content with the course pursued in either of the preceding, finding the hemorrhage terrific, and "not knowing what to do," he resorted to free "venesection." A remarkable remedy with which to overcome the exhaustion consequent upon excessive uterine hemorrhage. Notwithstanding this superadded loss of blood, the woman survived. Can there be any difficulty, Mr. President, in accounting for the mortality in the cases related by Dr. Gay? It is only wonderful that any could have survived.

Dissatisfied with the result of the practice pursued in the foregoing cases, the doctor concluded that "meddlesome midwifery was bad midwifery," and determined thereafter to "leave all to nature," simply inserting an "air-ball" into the vagina to lessen the hemorrhage. The wisdom of his course is demonstrated by lessened mortality in the cases which follow in his report, and in which little or no effort was made to interfere with nature in this *unnatural* position of the placenta.

Thus, Mr. President, does Dr. Gay propose to revolutionize the practice in all cases of placenta previa, and instead of making all cases, or nearly all artificial, as is the established practice, he would by his "new" course leave all these labors to nature, unaided. The course which I have thought, and taught, as the best to pursue, and to which opinion I must still adhere, notwithstanding the lucid arguments of the honorable member to whose paper we have just listened, is briefly this: that all cases of placenta previa should be treated as *artificial* from the moment the placenta was ascertained to occupy the uterine outlet, whether partially or completely, no matter whether labor came on at the full period of utero-gestation, or anticipated that time by a few weeks, as not unfrequently happens. Postponing delivery if the hemorrhage does not demand earlier interference, to the completion of gestation; at the commencement of the labor, and if possible, before much blood has been lost, *effectually* tampon the vagina and wait *only* until the uterine orifice is dilatable by gentle distension, and then at once proceed to turn and deliver, taking measures to secure uterine contraction at the same time.

As to the kind of tampon which the doctor recommends for the purpose of controlling the hemorrhage, it is not so good as soft, old muslin, torn into small slips and united by candle-wicking or tape, and then introduced through a cylindrical speculum. The latter can be made, if carefully introduced, and packed, to fill the vagina more completely than the ball, making more pressure upon the lateral aspects of the uterine outlet, and should fill the vagina thoroughly, so as to form a cylinder extending down to the os externum. Then making pressure upon the uterine tumor, over the abdomen, by a bandage or by the hand of an assistant, and applying a T bandage or a napkin held by a nurse to the external orifice of the vagina, pressure is made upon this cotton column which completely controls the bleeding. There is an additional advantage in the muslin tampon over the "new" one, or at least such it seems to me; it absorbs more the fluid or serous parts of the blood, and promotes the formation of coagula, and thus contributes to the arrest of hemorrhage.

Having thus protested against the adoption of the course recommended in the doctor's paper and briefly alluded to the orthodox mode of treating cases of placenta previa, I will not longer trespass upon the time of the Society.

Dr. Rochester said, the subject is a very important one, and I would like to occupy more time in the discussion than I can now spare. Have seen a large number of cases of midwifery during my professional life, but have never had but one case of placenta previa occurring in my own practice. Have seen two or three cases in consultation. It occurred to me that in one of the cases related by Dr. Gay, death might have occurred from rupture of the uterus. The fact that considerable force was used in the delivery and the post partem phenomena mentioned, lead me to suspect that there may have been rupture of the uterus. I am quite surprised that forcible delivery was resorted to in any of the cases mentioned, and am also surprised that ergot was not used in any of the cases.

Dr. Miner said that whatever might be his surprise at the treatment adopted in the first cases related, or proposed for future instances of placenta previa should they occur, it could not equal his astonishment at the statement that six cases had been met by any one physician in private practice, in the short space of eight months. He had now practiced his profession over twenty years, and had during all this period been as largely engaged in obstetrical practice as almost any of his associates, and as often called in cases of unnatural labor, or those requiring operative interference, and in his recollection had met with but a single case, and that not in his own practice. He could not state the exact proportion of cases as reported by authors, but it could not be greater than 1 in 2000. One case in the practice of either Dr. Gay or Hauenstein would be the full average; if they have by accident seen two or three during their professional lives, it would be greatly over the usual percentage. To expect the profession to accept the declaration that one of these physicians has treated six cases in eight months is too much; it can hardly be credited; it must be looked upon as a mistake; to think it, when there are so many sources of error is almost impossible. We must first conclude that the usual course of nature has been reversed, and that the placenta is being placed over the os uteri almost as a rule, rather than as a rare, very rare exception. The statistics of authors and the experience of the profession will not admit of such conclusion—it will appear much more likely that a physician, however experienced and capable, is mistaken in his diagnosis. The only motive in suggesting doubt as to the point of frequency consists in a desire to make the record of our reported cases as perfect as possible, and to show that we are not insensible of the influence statistics of disease may have upon the opinions and practice of others.

Dr. T. T. Lockwood said, that in the course of a practice of thirty years he had attended two thousand cases of midwifery. Of these, there were three cases of placenta previa, one of which was of central implantation; the other two were of lateral implantation, and the diagnosis in these cases was not positive; it was judged by the profuse hemorrhage and general symptoms. In the case of central implantation the patient died. Both the other cases terminated favorably. The head presented in every case. Turning was resorted to as soon as the os was easily dilatable. The use of ergot was resorted to early. Was greatly surprised to hear of so many cases occurring iu the practice of one practitioner in so short a space of time, and surprised that so great a degree of force was used in delivery.

Dr. L. also stated that of the two thousand cases attended by him he had used forceps in sixteen cases, perforated in two cases, and resorted to turning in twenty cases; a majority of cases of turning were of arm presentations. Has had twenty-six cases of puerperal convulsions, and been quite unfortuate in these cases, having lost about fifty per cent.

Dr. Gay remarked, in reply to the gentlemen who take exceptions to the views offered in the paper read, that these papers which are read before the Society, are of themselves perhaps but of little value, but the discussions that grow out of them are of very great value, and should always be conducted in a courteous manner. The cases of placenta previa reported to-night, are cases transcribed from my note-book, and the few observations made at the conclusion of the reported cases were such as the few leisure moments permitted me to make just previous to entering the room of the Association.

The central idea intended to be brought out by the reporter was simply this: that *delay* in placenta previa, until *complete* dilatation of the os, is justifiable; that if hemorrhage can be controlled, as we assume it may be, by the introduction of the air-ball, until complete dilatation, that delay is advisable, and that forcible entrance of the hand of the accoucheur through a rigid and undilated os, and the turning and delivery of the child, is more fatal to both mother and child, than to delay the efforts of art until nature had accomplished all she could toward the delivery by the full and complete dilatation of the os.

These views are sustained by the evidence furnished in the first case reported. Here was a woman in labor with placenta previa lateralis, for two days. According to the report this woman was under the care of a midwife when Dr. Storck and myself made our first visit. She lived fifteen days after her delivery, notwithstanding this delay, and in the absence of the appropriate means for the suppression of hemorrhage, for a period of two days previous to our visit. It is not unreasonable to think that possibly physicians make too great haste in their intermeddling, not only when treating placenta previa, but also when treating a case of puerperal convulsions. It is not unreasonable to think that perhaps women in either of these perilous situations may have been sacrificed to hasty interference.

I am certainly not an advocate of venesection in placenta previa; should not advise it, and probably shall never have occasion to resort to it again, and probably should not have recourse to it if I had occasion, but in the case reported, occurring in my own practice some years ago, when venesection was more fashionable than now, it were folly to say that the end did not justify the means. Venesection in unavoidable hemorrhage when this case occurred, received the sanction of the highest authority.

The third case of placenta lateralis was merely referred to as a suggestive case. It suggests the idea that there are other causes beside actual hemorrhage which are at work conspiring toward a fatal result. I have the authority of Dr. Hauenstein for saying that no more blood was lost, or not so much even, as we were formerly accustomed to abstract from the arm for pneumonia, or no more than is lost in an ordinary normal labor.

It was the purpose of the reporter to call attention more especially to the three cases of central implantation, in the management of which I was associated with my friend, Dr. Hauenstein, from beginning to end. In the first case there was no delay; the os was not fully dilated by the efforts of nature, but was forcibly dilated by the hand of the accoucheur; death of the woman occurring soon enough thereafter to satisfy the claims of any one who may be an advocate of the doctrine of no delay. If I may have used here to-night the term forcible delivery, I mean just this, and no more, viz: the forcible entrance of the hand within an undilated and rigid os.

Of the other two cases, in spite of an almost uncontrollable desire to deliver these women at once, to get them off our hands and to go home to bed and rest, non-interference was advised and complied with, until the os was completely dilated, and the result was safety both to the mothers and their offspring.

The notable fact which I have stated, that Dr. Hauenstein had seen six cases of placenta previa during the last eight months, seems to be received with some incredulity, or to state the case more concisely, is denied in toto. Leaving out of the discussion entirely the question of my own truth and veracity, permit me to say of Dr. Hauenstein, that he is a practitioner of twenty-five years, and that he is a gentleman. He enjoys a large obstetric practice, is called by a dozen or more midwives in as many different directions in the city, and always in difficult cases. If there be any other man more reliable in character for truth and veracity, show me the man. Dr. H. was called to these cases and diagnosed them as reported, and his diagnosis was confirmed by myself, therefore for gentlemen here this evening who saw not the cases at all, to cast any shadow of doubt upon the diagnosis is simply absurd and unprofessional.

The first case of central implantation died from post-partem flooding in an hour and a half. Two physicians examined the woman and tell you she had placenta previa. Do you doubt the correctness of the diagnosis? Hemorrhage occurred in the other two cases at eight months; the position of the child in either case was transverse, which position is common in placenta previa; their labors commenced and ended with hemorrhage, and two physicians on examination pronounced them to be cases of placenta previa. Can you doubt the correctness of the diagnosis? If you still doubt, then let me add the evidence of the sense of sight to that of the sense of touch. After detachment of the placenta they were minutely examined, and the presenting portion was clearly recognized and separated from the portion which did not present; but my friend on my left, Dr. Miner, who at first positively stated his doubts of the truthfulness of the statement of Dr. Hauenstein, now disclaims any intention of calling in question the truth and veracity of the doctor, but thinks the doctor and myself in error. Dr. White, also, does me the justice to say that he does not call in question our veracity, but thinks an error has been committed. Gentlemen, I accept of no such version of the subject matter. I repel it; there was no error; there was no mistake committed; it was impossible to commit error, and the diagnosis was correct beyond the shadow of a doubt, and there is no profit in caviling over a question from which no good can be derived and contributed to science.

We all agree in belief as to the extreme peril of the woman in child-birth with placenta previa. Twenty years ago I remember to have heard Dr. Meigs say that he had rather take the chances of his life with a loaded pistol, pointed directly at his head, at the distance of five paces, and fired off with deliberate aim, than take the chances of life of the woman with placenta previa; and he facetiously added that he had rather be up a gum-tree than called to a case. It is because of this extreme peril that has led my friend, Dr. White, to say there should be no delay. I do not misquote him here, although misquoting the doctor in the use of the word "forcible," for I have written down his words as they were uttered. These words have led me to suppose that he was in favor of immediate delivery so soon as the hand could be pressed forward through a rigid os, the child seized and delivered; but I am happy to stand corrected, and to say that I have misapprehended the doctor, who, although deprecating delay, is favorable to the plan of waiting until the os is *dilatable*. He who acts upon this plan acts rightly and wisely. When labor has so far progressed that the os is dilatable, or what is synonymous to this, when the os is completely dilated, then nature can accomplish no more. She should be left to her own unaided efforts up to this point, beyond which she cannot go; her task is completed; then art comes in to her aid, and any delay beyond this point would be inexcusable.

Dr. Miner replied, that Dr. Gay must accept the statement as it was, both for himself and Dr. Hauenstein. It was only claimed that error in diagnosis was much more probable than the occurrence of the cases. He did not question the veracity of any one, but remembering the sources of error—the difficulty of determining the place of implantation when situated laterally or posterially, the liability of firm coagulum filling the os and lower portion of uterus, and of a detached placenta to descend and present in front of the child, he had expressed his doubts, but not with any intention of intimating otherwise than possibility—probability of error in diagnosis.

Dr. Ring had been interested in the paper read by Dr. Gay. The number of cases reported was *extraordinary*. To make a correct diagnosis in placenta previa was not difficult; could not think there had been a mistake in this respect; had implicit confidence in any matter of fact that Drs. Gay or Hauenstein might report. Obstetric practice among our German population was almost entirely in the hands of midwives, and they called on physicians as a dernier resort. It followed that our German practitioners had a much larger proportion of dangerous and difficult labors under their care than the average of the profession. It was probably the experience of all present that occasionally one labor after another would be unnatural and complicated. We frequently see in quick succession diseases which occur as rarely as placenta previa. The members of this Association will remember that within a few months several cases of pelvic hæmatocele were reported here. Had been in practice over twenty years. Had attended about a thousand cases of labor. Placenta previa had occurred but once in his practice. Prof. White, who saw this case in consultation, will remember that our patient died after delivery by turning, from post partem hemorrhage. To suppress hemorrhage after delivery, we gave ergot, applied cold, made pressure with the hand, and at last stimulants, which he thought correct practice. Regarded it as dangerous to delay delivery after the uterus was dilatable.

The profession had been familiar with the able essay of Prof. Simpson of Edinburgh, and the line of practice proposed by him. He enquired of those present if they had ever treated any cases in the manner proposed by Prof. S., and if so, with what result? or, if it would be justifiable with our present experience to resort to that mode of treatment?

Dr. White said, my friend, Dr. Ring, inquires as to the treatment of Radford and Simpson, which attracted a good deal of attention a few years since, and which has now passed into disuse. The hypothesis upon which it was recommended by these gentlemen to completely detach the placenta, and then leave the labor to nature, except hastening delivery by the administration of ergot, etc., assumed that the blood in this form of hemorrhage came from the vessels made patulous in the *placenta* by its partial detachment, and not from the uterine orifices thus laid open. It is difficult to determine how large a proportion of the blood comes directly from the open uterine vessels and how much from the ruptured placental vessels. It is certain that the uterine orifices bleed freely until contraction can take place and close them, therefore the less the surface separated before delivery the less the danger to the woman from hemorrhage. That *fatal* hemorrhage *may* occur after delivery of the child and placenta, when the blood can only be discharged from the uterine vessels is well known, and was demonstrated indeed in a case of placenta previa occurring in the practice of himself, (Dr. Ring) in the treatment of which I was associated. In the case referred to the woman was very comfortable after delivery, and subsequently sunk, notwithstanding all our efforts to arrest it, from post-partem hemorrhage. This case alone is sufficient to disprove the assertion of the advocates of this doctrine, that hemorrhage is arrested by completely detaching the placenta from its connection with the uterus. Indeed there is great doubt whether the safety of the mother is promoted by thus severing the utero-placental connection in the early stage of labor, and it is conceded by all that the child is doomed to certain destruction, and the practice is therefore pretty nearly abandoned. In an interview with Sir James Simpson, who has done more to bring this mode of procedure into favorable notice than any other man, he remarked that he "now resorted to it less frequently than formerly, and only in exceptional cases."

I regret to hear any of the members lay so much stress upon the extraordinary fact that so many cases should have occurred in so short a period in the practice of an individual. The frequency of their occurrence bnt increases the importance of adopting correct views in their management. There can be no question of veracity entertained, and the principles governing the treatment is the same, whether they occur in every first or in every second thousand. In an extensive obstetrical practice extending over thirty-six years, and during the last twenty having a large number in consultation with my fellow practitioners, I have seen but nineteen cases of placenta previa. Four of these only occurred in my own private practice, the remainder were in the hands of others, (several of whom I see present,) living in the city or its vicinity. Two of the nineteen only proved fatal to the mother. The practice pursued has invariably been to turn and deliver at the earliest period compatible with safety to the mother's tissues, and during the last twenty years administering chloroform or some other anæsthetic while performing the operation of turning.

One word more, Mr. President, and I have done. Dr. Gay seems to have become so confused that he entirely misapprehends the plainest statements. As all the members present are aware, I have, in my remarks, used the word "forcible" *only* to protest against the use of *force* under all circumstances. Again, how he can get himself into such a muddle in relation to my use of the word *delay*, for I insist I did not say "*no* delay," when, as you all know, I distinctly stated that he should, in all cases, *delay* until the mouth of the uterus was dilated, or dilatable with gentle effort, at which time, but not sooner, the accoucheur should proceed to turn and deliver. I *insist*, Mr. President, on being correctly quoted upon so important a question.

Dr. Green said he was sorry that Dr. Hauenstein was not present to report the cases mentioned by Dr. Gay. Have no doubt but these cases may have been seen. In the location in which I practice there are many midwives, and I am often called in difficult cases that have been attended by midwives in the early stages of the labor. Have seen two cases of placenta previa in a practice of sixteen years. The mother and child have been saved in both cases. Turned and delivered in both cases as soon as the os was sufficiently dilated. Although the number of cases reported as occurring in the practice of Dr. H. is quite remarkable, still I think they may have occurred.

Dr. Diehl said, I have had but few cases of midwifery, but have nearly always had difficult cases, and believe that it may and often does occur that practitioners have a succession of difficult and remarkable cases. It is the custom among the German people to employ midwives and send for help when they have difficult cases. This fact probably accounts for the large number of these cases seen by Dr. Hauenstein.

Dr. Eastman said, have been in practice fifteen years, and have had fully my share of midwifery, and have had but two cases of placenta previa. Have never known so many cases reported by any practitioner. It is not only remarkable, but *remarkably* remarkable, that so many cases should occur in the practice of any practitioner within so short a time.

Dr. White called for the reading of the By-Laws in regard to dues and membership, and moved that said By-Laws be enforced. Carried.

Scarlatina, pneumonia and rheumatism were reported as prevailing.

Adjourned. T. M. Johnson, Sec'y.

